# Spatiotemporal distribution of community-acquired phenotypic extended-spectrum beta-lactamase *Escherichia coli* in United States counties, 2010–2019

**DOI:** 10.1017/ice.2023.266

**Published:** 2024-04

**Authors:** Matthew W. Smith, Margaret Carrel, Qianyi Shi, Shinya Hasegawa, Gosia Clore, Zhuo Tang, Eli Perencevich, Michihiko Goto

**Affiliations:** 1 Center for Access & Delivery Research and Evaluation (CADRE), Iowa City Veterans’ Affairs Health Care System, Iowa City, Iowa; 2 Division of Infectious Diseases, Department of Internal Medicine, University of Iowa Carver College of Medicine, Iowa City, Iowa; 3 Department of Internal Medicine, University of Iowa Carver College of Medicine, Iowa City, Iowa; 4 Department of Geographical and Sustainability Sciences, College of Liberal Arts and Sciences, University of Iowa, Iowa City, Iowa

## Abstract

Using data from the Veterans’ Health Administration from 2010 to 2019, we examined the distribution and prevalence of community-acquired phenotypic extended-spectrum β-lactamase (ESBL) *E. coli* in the United States. ESBL prevalence slowly increased during the study period, and cluster analysis showed clustering in both urban and rural locations.

In the United States, bacteria that are resistant to antibiotics cause >23,000 deaths each year and are responsible for >2 million infections annually, with >$20 billion in related spending every year.^1^ Current knowledge of bacterial resistance patterns in the United States is dependent on nonspatial surveillance data, meaning that spatial patterns of resistance are poorly understood.

*Escherichia coli* has increasingly developed resistance to β-lactam antibiotics, and isolates that produce extended-spectrum β-lactamase (ESBL) have become a serious concern due to their broad resistance profiles and increased patient mortality.^2,3^ The global prevalence of ESBL *E. coli* is rising, having increased from 2.6% to >20% over the past 20 years.^3,4^ ESBL prevalence varies by region, however, highlighting the need to better understand factors that influence antibiotic resistance. In this study, we focused on outpatient resistance patterns in the United States, particularly on community-acquired phenotypic ESBL *E. coli,* which we defined in this study as isolates intermediate or resistant to ceftriaxone, cefotaxime, or ceftaroline.

## Methods

We identified patients who received care in the outpatient environment of the nationwide Veterans’ Health Administration (VHA) between January 1, 2010, and December 31, 2019. All patients with at least 1 clinical isolate of *E. coli* from any site were included, but we excluded isolates that were sampled at institutionalized settings (eg, inpatient, nursing home). The study population of the VHA includes residents from all 3,108 counties within the continental United States and the District of Columbia who received care at any of the 168 hospitals and 1,085 outpatient clinics in the VHA.

Clinical data were extracted from the VHA Corporate Data Warehouse. No information was available on bacterial genetics or mechanisms of resistance, including ESBL-specific test results. Samples were excluded if they were collected >48 hours after admission to an institutionalized setting (ie, acute care, long-term care, or mental health unit). Isolates were assigned to counties based on geocoded residential addresses of individual patients, and only the first isolate for each patient in a calendar month was included in the analysis.

The primary outcome measure was annual county-level phenotypic ESBL rates among *E. coli*. Nationwide and county-level yearly phenotypic ESBL proportions were calculated using descriptive statistics. County-level rates were assigned to the spatial centroids of counties using Federal Information Processing Standard codes. Analyses were performed with R version 3.5.2 software)^5^ using the *ggmap* package. Choropleth maps were generated using ArcMap version 10.5 software.^6^ Cluster analyses were performed using SaTScan version 0.3.92 software,^7^ specifically space–time analysis with Poisson probability model, scanning only for high rates of clustering with 3-month time aggregations, using a maximum of 20% of the population at risk with circular spatial windows and maximum of 50% of the study duration.

The Institutional Review Board of The University of Iowa and the Research and Development Committee at the Iowa City Veterans’ Affairs Health Care System approved this study and granted a waiver for informed consent for this retrospective observational study with minimal risk.

## Results

**Figure 1.**
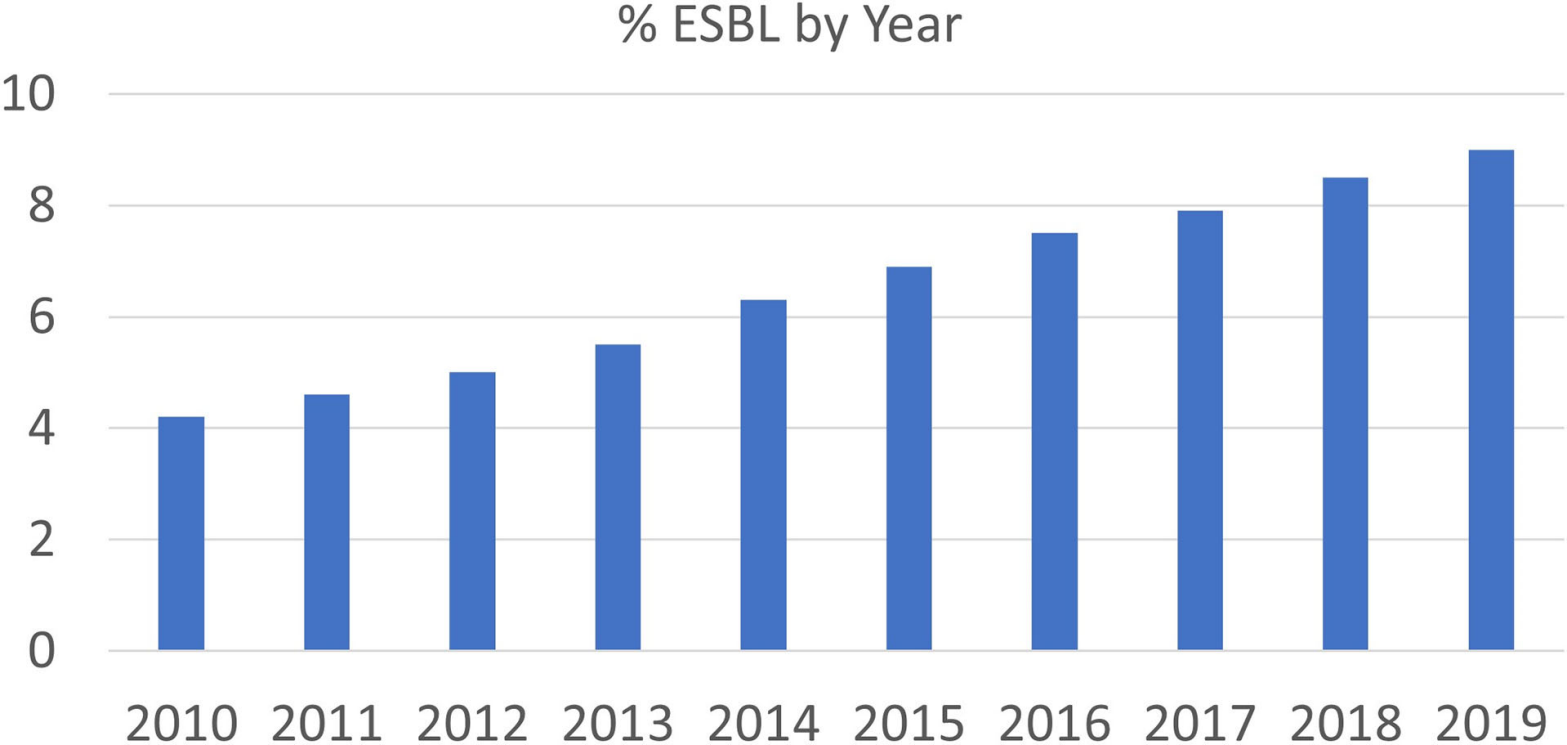
Percentage of isolates with ESBL phenotype by year.

From 2010 to 2019, 625,562 unique clinical cultures grew *E. coli*. Of these, 41,838 (6.7%) met the study definition of phenotypic ESBL organism. The national proportion of phenotypic ESBL *E. coli* isolates acquired in the community increased from 4.1% in 2010 to 9.0% in 2019 (Fig. 1).

**Figure 2.**
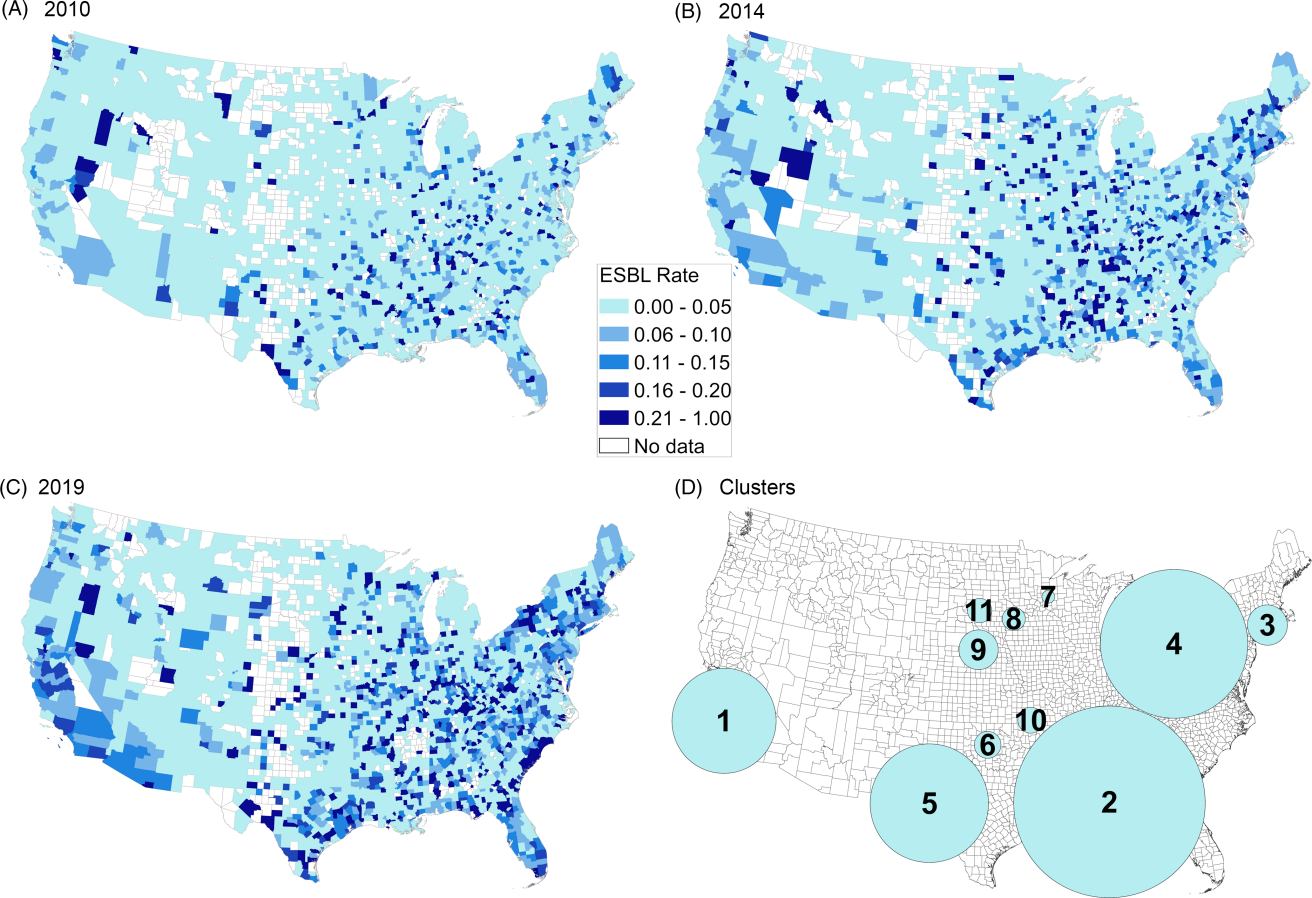
Percentage of isolates with ESBL phenotype by county in 2010 (A), 2014 (B), 2019 (C). Cluster analysis in panel D.

Figure 2 shows the proportion of phenotypic ESBL isolates by county during selected years (2010, 2014, and 2019), and cluster analysis results. In 2010, most US counties had low levels of ESBL *E. coli* (0–5% ESBL), with only a few geographically noncontiguous counties exhibiting high levels of resistance (>20% ESBL). Over time, an increasing number of counties exhibited rates of ESBL of >20%.

Overall, 11 spatiotemporal ESBL *E. coli* clusters were identified. These clusters represented an area where county centroids had significantly higher numbers of ESBL isolates than expected when compared to the surrounding regions (Supplementary Table 1 online). These clusters ranged greatly in size and were located in many parts of the county. The clusters with the highest log-likelihood ratios (LLRs) were centered in southern California, southern Alabama, Connecticut, and southern Ohio. Relative risk among the clusters ranged from 1.37 (cluster 4) to 5.26 (cluster 11). All 11 clusters had a temporal range of at least 1 year, and 9 of the clusters occurred in the second half of the study period, whereas only 2 clusters (clusters 7 and 11) occurred during the first half of the study period.

## Discussion

In this nationwide spatiotemporal analysis using data from the VHA, we identified increasing prevalence of community-acquired phenotypic ESBL *E. coli* as well as heterogenous spatial distribution of resistant isolates. The slowly increasing rates of phenotypic ESBL *E. coli* found in our study are consistent with recent data from the United States.^8^

We identified 11 clusters of increased community-acquired ESBL *E. coli* prevalence across the county. We theorize that the overall higher antibiotic usage in large population centers is responsible for the clusters of ESBL *E. coli* observed in the highly populated areas of southern California, the northeastern United States, and the Gulf Coast states. However, several clusters of resistance occurred in regions without major urban centers. This raises the question of which community-level factors outside human antibiotic consumption are contributing to antibiotic resistance, for example, antibiotic use in livestock. ESBL rates in European Union livestock have been previously reported as 1.6% (poultry), 2.3% (hogs), and 8.5% (cattle).^9^ These rates have been attributed to the liberal use of third- and fourth-generation cephalosporins in animals.

The clustering of ESBL *E. coli* infections in rural areas in the United States suggests that antibiotic usage in livestock might be contributing to human ESBL infections. For example, the high-risk cluster in southwest Minnesota–northwest Iowa is the largest hog-farming region in the country, and hogs are also substantially present in eastern North Carolina, which exhibited ESBL resistance prevalence of >20% during several years of our study. Our study findings highlight the need to further explore the connection between humans, animals, and their shared environment, an approach consistent with the ‘One Health’ movement.^10^

A key strength of this study is the large, national data set of VHA patients spanning a 10-year period, which led to a robust data set at the county level which is otherwise challenging to obtain in the United States. Secondly, inclusion of all *E. coli* isolates increased our ability to capture the spectrum of *E. coli* disease activity.

This study had several limitations. Large areas in the central United States had no data points due to the small patient population in those areas. This factor may have masked relevant spatial relationships. Cluster mapping is an exploratory method of data analysis and is not designed to make causal inferences. Also, genetic ESBL testing would have improved our understanding of antibiotic resistance patterns, but these data were not available for our study. Finally, we looked exclusively at outpatient ESBL isolates, which may not have reflected inpatient ESBL infection trends.

These findings highlight the increasing prevalence of community-acquired phenotypic ESBL *E. coli* over time as well as the spatiotemporal distribution of this organism. Cluster analyses showed areas of significant clustering in both urban and rural locations, suggesting that further research is needed to elucidate the population and environmental drivers of resistance, such as time-series analyses of temperature, climate, and population trends.

## Supplementary material

To view supplementary material for this article, please visit https://doi.org/10.1017/ice.2023.266
